# Characterization of Five *Meloidogyne incognita* Effectors Associated with PsoRPM3

**DOI:** 10.3390/ijms23031498

**Published:** 2022-01-28

**Authors:** Wenjiang Pu, Kun Xiao, Sifang Luo, Haifeng Zhu, Zizhen Yuan, Chaoyuan Gao, Jianfang Hu

**Affiliations:** Laboratory of Fruit Physiology and Molecular Biology, China Agricultural University, Beijing 100193, China; puwj07@outlook.com (W.P.); xiaokun201@outlook.com (K.X.); Sifang.Luo@outlook.com (S.L.); zhf51@outlook.com (H.Z.); yzznbst@outlook.com (Z.Y.); cristianogao7@outlook.com (C.G.)

**Keywords:** Xinjiang wild myrobalan (*Prunus* *sogdiana*), *Meloidogyne incognita*, resistance, effector, function

## Abstract

Root-knot nematodes (RKNs) are devastating parasites that invade thousands of plants. In this study, five RKN effectors, which might interact with *Prunus* *sogdiana* resistance protein PsoRPM3, were screened and identified. In situ hybridisation results showed that MiCal, MiGST_N_4, MiEFh and MiACPS are expressed in the subventral oesophageal glands (SvG), and MiTSPc hybridization signals are found in the dorsal esophageal gland (DG) of *Meloidogyne incognita* in the pre-J2. RT-qPCR data indicated that the expression of *MiCal*, *MiGST_N_4*, *MiEFh*, and *MiACPS* genes are highly expressed in *M. incognita* of pra-J2 and J3/J4 stages. The expression of *MiTSPc* increased significantly in the female stage of *M. incognita*. Moreover, all effectors found in this study localize in the cytoplasm and nucleus when transiently expressed in plant cells. In addition, MiGST_N_4, MiEFh, MiACPS and MiTSPc can elicit the ROS burst and strong hypersensitive response (HR), as well as significant ion leakage. Our data suggest that MiGST_N_4, MiEFh, MiACPS and MiTSPc effectors may be involved in triggering the immune response of the host plant.

## 1. Introduction

Root-knot nematodes (RKNs; *Meloidogyne* spp.) are among the biggest threats to agricultural production around the world, causing over $80 billion of losses annually [1,2]. Tremendous numbers of crops and trees are seriously invaded by root-knot nematodes [3,4,5]. RKNs infect the roots of crops and hinder the absorption of water and nutrients, leading to retardation and reduction in growth and development, resulting in a decline in yield and quality [6]. During parasitism, root-knot nematodes deliver nematode secretions (effectors) to plant cells via their stylets, suppressing plant defenses and inducing and/or maintaining feeding sites, i.e., the giant cells (GCs) [7]. Multinucleated cells developed by giant cells through intranuclear replication are the only source of nutrients for the growth and development of root-knot nematodes [2,8,9]. The development of nematodes from eggs to adults requires several different effectors to ensure their parasitism and life cycle in the host [10]. Currently, several nematode effectors have been identified by proteomic analysis, transcriptome and genome sequencing, as well as bioinformatics approaches [11,12,13]. However, the function of most effectors remains unclear. The function for many effectors has still not been elucidated.

Recent studies have shown that when RKNs penetrate and migrate into the root, they secrete effectors related to degradation or modification of the cell wall [14,15]. In addition, another important role for effectors is to promote the formation of feeding sites and giant cell to obtain adequate nutrient supply [16]. Therefore, to favor nutrient supply required for growth, RKNs secrete effectors to hijack the host’s development and metabolism [17,18]. In the parasitic stage, effectors that inhibit plant immune responses to against plant defense responses or to protect nematode feeding cells are secreted by RKNs [19,20,21]. In addition, some effectors can help RKNs evade the attack of the plant immune system [22]. Therefore, the most important function of effectors is to suppress the host immune response. The different stages of root-knot nematode infection, such as initiating invasion, establishing feeding sites and inducing giant cell formation, require a large number of functional effectors [23,24]. However, it is still unclear which functional effectors are required by nematodes at specific times in different parasitic stages and in different tissue cells.

In *Meloidogyne incognita*, the effectors Mi-gsts-1, MiISE5, Misp12, and MiMsp40 are specifically expressed during the period of invasion and act in critical roles to overcome host defense responses and promote parasitism [25,26,27,28]. The *M. incognita* effector MiEFF18 can interact with essential components of the spliceosome SmD1, a complex involved in pre-mRNA splicing and alternative splicing in host plants. MiEFF18 modulates SmD1 to facilitate giant cell formation [29]. Effector MiEFF1 targets cytosolic glyceraldehyde-3-phosphate dehydrogenases (GAPCs) that are induced in giant cells, and their nonmetabolic functions are required for root-knot nematode infection [30]. In addition, some *M. incognita* effectors can promote relative invasion and parasitism by specifically interfering with functional proteins involved in plant defence, such as the effectors MiMIF-2, MiISE6, and Minc17998 [31,32,33]. These studies have revealed the functions of some nematode effectors and indicated complex interactions between nematodes and host plants.

In plant-pathogen interactions, the famous ‘Z’ model has accurately interpreted changes in the host defensive response [34,35]. According to this model, plants have evolved two layers of immunity: PAMP-triggered immunity (PTI), and effector-triggered immunity (ETI). During both PTI and ETI, plants rapidly produce large amounts of reactive oxygen species (ROS), an important signaling component in metabolic and stress responses in plants [36,37,38,39]. ROS production has been considered as one of early events of plant defense signaling [40,41]. Another effective strategy against the pathogen is known as HR, which generally occurs at the infection sites [42,43]. Plant resistance genes also play an important role in this process. Currently, some resistance genes have been reported such as *H1*, resisting potato cyst-nematodes in potatoes, *Hsl^pro-1^* against *Heterodera schachtii* in sugar beet, *Mi-1* resisting *M. incognita* in tomato, and *Ma* resisting *M. incognita* in French cherry plum. [44,45,46,47]. However, an exact molecular model of nematode-plant interactions regulating PTI and ETI has not been established. Recently, research has clarified that ROS accumulation induced by PAMP contributes to enhancing the intensity of ETI and HR cell death [38]. In turn, sustained ROS induced by ETI can trigger a strong PTI-ROS burst [48].

Previously, we cloned and identified an NBS-LRR-type disease resistance gene *PsoRPM3* from the RKNs-resistant plant *Prunus* *sogdiana* [49]. Here, 17 nematode effector candidates (Supplementary Table S1), which have the potential to target PsoRPM3 and influence plant resistance, were obtained by performing immunoprecipitation (IP) and liquid chromatography-tandem mass spectrometry (LC-MS) analysis in *P. sogdiana*. Five *M. incognita* effector proteins were selected to clarify the character and function in the 17 candidate nematode effector proteins. In situ hybridization was performed to analyze the spatial expression of these effectors in *M. incognita.* The developmental expression pattern of these effector genes was analyzed by RT-qPCR. *Agrobacterium*-mediated transient expression in disease-resistant tobacco showed that four effector proteins can induce a burst of ROS and HR. Consistent with the visual observations, the four effector proteins significantly increased ion leakage levels compared to control. Our data preliminarily determined the functions of the four effectors from *M. incognita*.

## 2. Results

### 2.1. Five Candidate Effectors: Gene Amplification and Sequence Analysis

**Figure 1:**
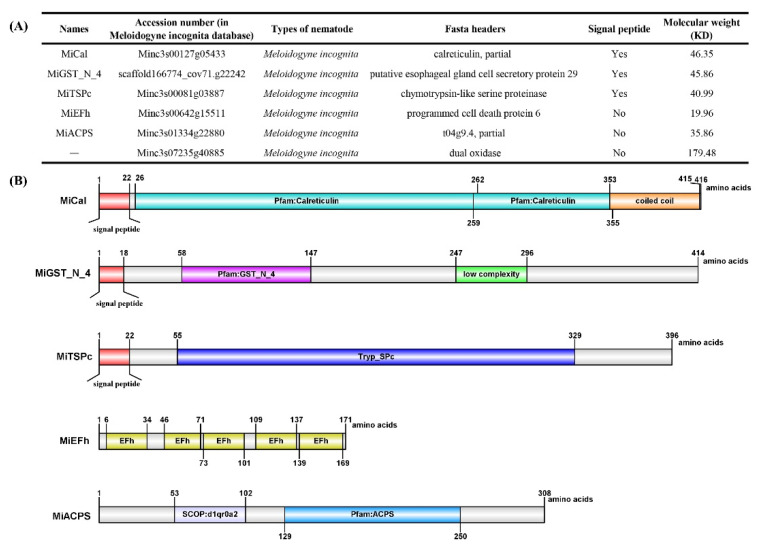
(**A**) Effector proteins from *Meloidogyne incognita* obtained by immunoprecipitation (IP) combined with liquid chromatography coupled with a tandem mass spectrometry (LC-MS) assay, showing their accession number in the *M. incognita* database, signal peptide status and protein molecular weights. (**B**) The characteristics of the five effectors of *M. incognita*.

We previously reported an RKN resistance gene, *PsoRPM3,* derived from *P. sogdiana* [49]. Immunoprecipitation (IP) combined with liquid chromatography coupled with a tandem mass spectrometry (LC-MS) assay was performed to screen for its *M. incognita* interactors (Supplementary Table S1). Six effectors (Minc3s00127g05433, scaffold166774_cov71.g22242, Minc3s00081g03887, Minc3s00642g15511, Minc3s01334g22880, and Minc3s07235g40885) were obtained from *M. incognita* (Figure 1A). Previous research reported that the molecular weight of effectors that can trigger plant immunity is generally smaller than the disease-resistance protein [50,51]. The molecular weight of the effector Minc3s07235g40885 obtained via IP and LC-MS is 179.48KD (Figure 1A), which is bigger than PsoRPM3 (107.490 KD). Therefore, Minc3s00127g05433, scaffold166774_cov71.g22242, Minc3s00081g03887, Minc3s00642g15511, and Minc3s01334g22880, but excluding Minc3s07235g40885, were cloned in *M. incognita* and their characters analyzed. The results revealed that *Minc3s00127g05433* cDNA encoded a 416 amino acid protein which contains two Calreticulin domains and a coiled-coil region, with a predicted molecular size of 46.35 kDa that consisted of an N-terminal signal peptide of 22 amino acids, named MiCal in this study. Scaffold166774_cov71.g22242 has 414 aa (amino acids) containing the GST_N_4 domain and an unknown (low complexity) region at position 58–147 aa and 247–296 aa, respectively, with an N-terminal signal peptide of 18 amino acids and was named MiGST_N_4. *Minc3s00081g03887* cDNA encodes a 396 amino acid protein with a predicted 22 amino acid N-terminal signal peptide for secretion and a Tryp_SPc domain, named MiTSPc, containing the predicted bacterial Ig-like domain 1 (1–6 aa) and TRYPSIN_DOM Serine proteases (55–329 aa) motif. The open reading frame (ORF) of *Minc3s01334g22880* contains 513 nucleotides, encoding a protein of 171 amino acids including five EFh domains, named MiEFh. The protein sequence of Minc3s01334g22880 contains SCOP: d1qr0a2s and ACPS domain, named MiACPS. Minc3s01334g22880 and Minc3s07235g40885 do not have signal peptides at the N-terminus (Figure 1B).

### 2.2. In Situ Hybridization in Pre-Parasitic Second-Stage Juveniles (Pre-J2) and Localization Analysis of Effectors

**Figure 2:**
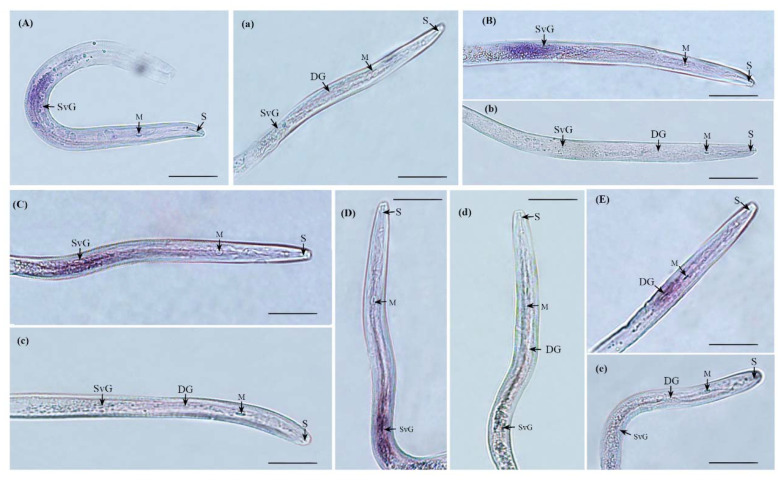
In situ hybridization of pre-J2 of *M. incognita*. (**A**–**E**) indicate the location of secretion detected by *MiCal*, *MiGST_N_4*, *MiEFh*, *MiACPS*, and *MiTSPc* antisense probes in second instar larval tissues of *M. incognita*, respectively. (**a**–**e**) indicate the detection of secretion location by *MiCal*, *MiGST_N_4*, *MiEFh*, *MiACPS*, and *MiTSPc* sense probes, respectively. Hybridization signals for all effectors were observed at the same secretory sites in at least 3–5 nematodes, and we showed one of the nematodes with hybridization signals for all effectors. Subventral oesophageal glands (SvG) and dorsal esophageal gland (DG), metacorpus (M), and stylet (S) are indicated with arrows. Scale bars, 50 μm.

To investigate the localization of effectors in *M. incognita*, in situ hybridization assays were performed. The specificity of all probe synthesis primers was detected by agarose gel electrophoresis (Supplementary Figure S1). A synthesized antisense strand probe and sense strand probe were used for in situ hybridization. The results showed that digoxigenin-labeled single-strand antisense cDNA probe of *MiCal*, *MiGST_N_4*, *MiEFh*, and *MiACPS* specifically hybridized in the subventral oesophageal glands (SvG) of pre-J2 of *M. incognita* (Figure 2A–D), whereas the *MiTSPc* transcripts were specifically expressed in the DG of pre-J2 of *M. incognita* (Figure 2E). As a negative control for the hybridization, a sense probe against effector was used, which did not induce a signal (Figure 2a–e).

### 2.3. All Effectors Localize in the Nucleus and Cytoplasm when Expressed in Nicotiana benthamiana Cells

**Figure 3:**
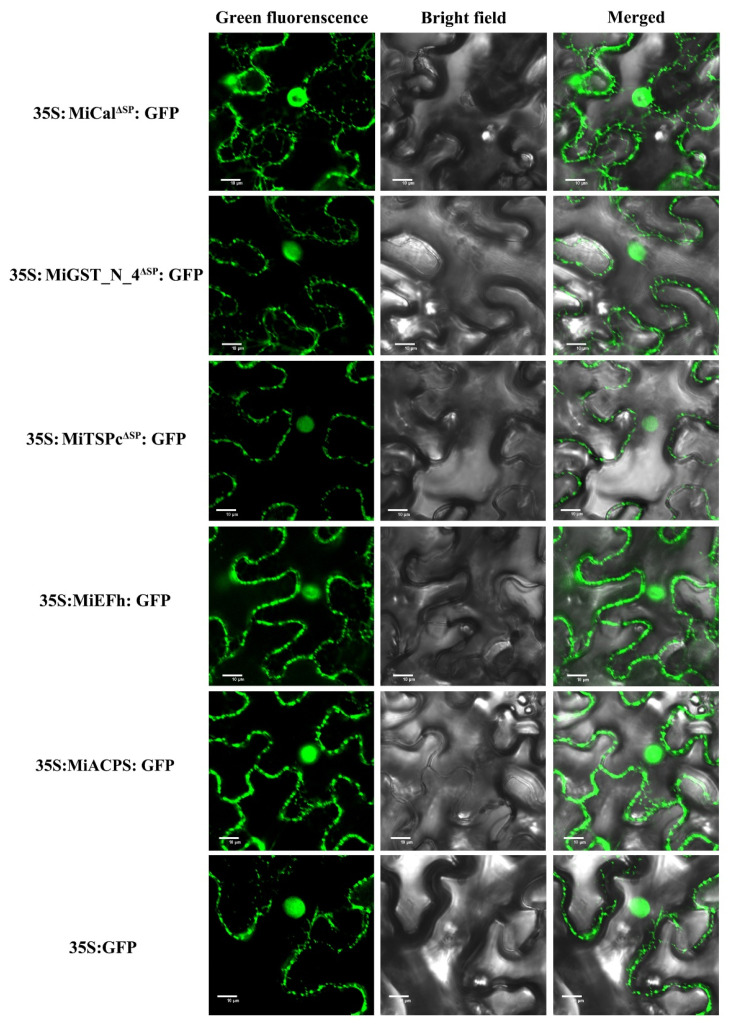
Subcellular localization of the five effectors when expressed in plant cells. *Agrobacterium* culture harboring the 35S: MiCal^ΔSP^: GFP, 35S: MiGST_N_4^ΔSP^: GFP, 35S: MiTSPc^ΔSP^: GFP, 35S: MiEFh: GFP, and 35S: MiACPS: GFP construct were hand-injected into WT *Nicotiana benthamiana* leaves at an inoculum of OD_600_ = 1. Epidermal leaf tissues were collected and subjected to microscopy with a confocal laser scanning microscope post infiltration 48 h. The experiment was conducted three times with similar results. Scale bars, 10 μm.

**Figure 4:**
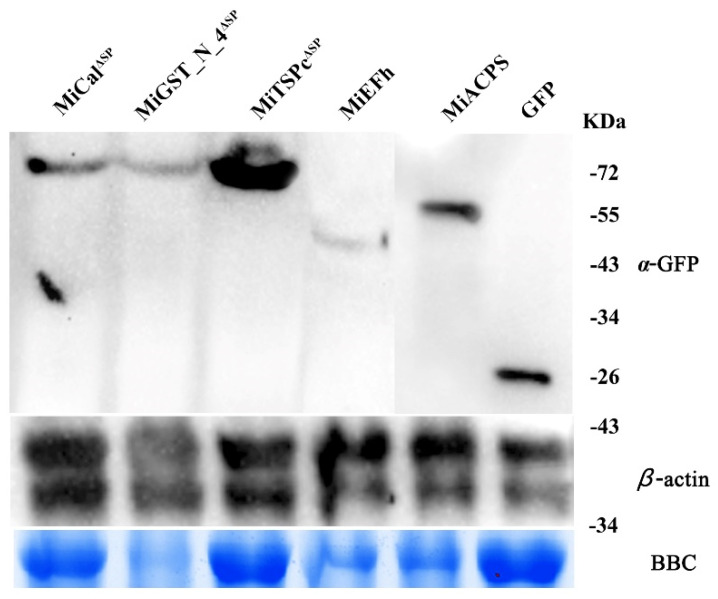
The normal expression and accumulation of all effectors in plant cells were analyzed by Western blotting. Protein detection used anti-GFP to detect the expression of effectors. The use of Coomassie brilliant blue (CBB) staining showed the loaded protein samples.

Most nematode effectors are delivered into a host cell and localized to specific subcellular compartments to perform the distinct activity [52]. The online analysis revealed that MiTSPc contains a putative nuclear localization signal, while MiCal, MiGST_N_4, MiEFh, and MiACPS do not. To verify subcellular localization, the expression construct of 35S: MiCal^ΔSP^: GFP, 35S: MiGST_N_4^ΔSP^: GFP, 35S: MiTSPc^ΔSP^: GFP, 35S: MiEFh: GFP, and 35S: MiACPS: GFP were transformed into *Agrobacterium tumefaciens* GV3101 strain and transiently expressed in leaf epidermal cells of *N. benthamiana*. The results indicate that all effectors were localized in the nucleus and cytoplasm (Figure 3). Western blotting results showed that they were normally expressed and accumulated in leaves cells (Figure 4).

### 2.4. Expression Pattern Analysis of Five Effectors in M. incognita

**Figure 5:**
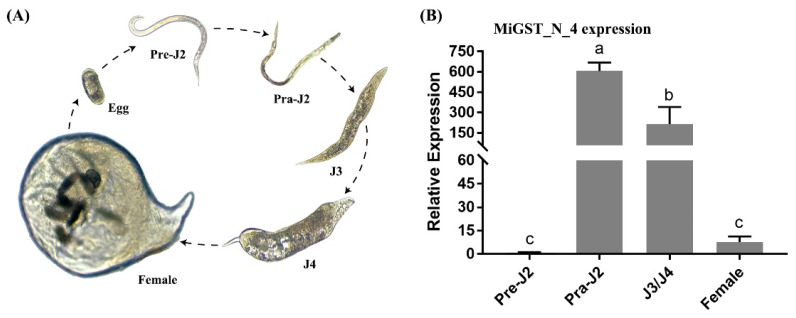

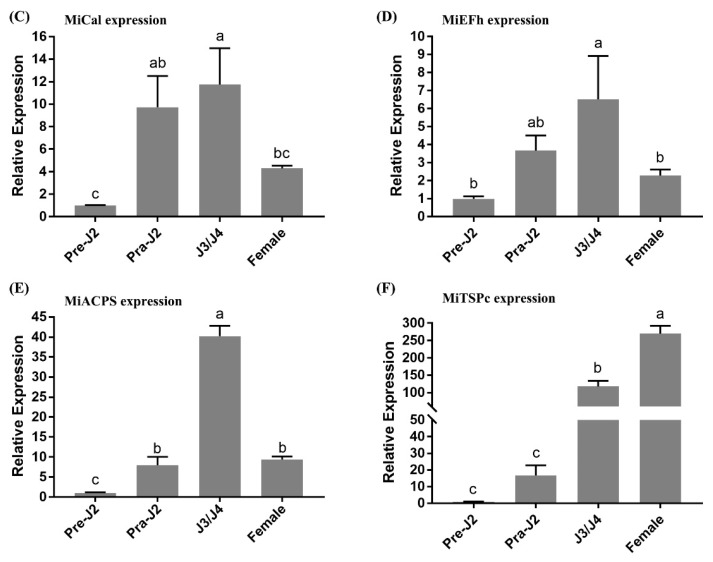
Developmental expression of the five effectors of *M. incognita*. (**A**) The growth cycle of *M. incognita*. “Egg” indicates eggs obtained from gall masses; “Pre-J2” indicates newly hatched pre-parasitic second-stage juveniles *M. incognita* from eggs; “Pra-J2” indicates parasitic second-stage juveniles collected from the RKN-susceptible tobacco variety W38 after inoculated with Pre-J2 *M. incognita* for 7 days; “J3/J4” indicates parasitic third-stage juveniles (J3)/ parasitic fourth-stage juveniles (J4) collected from the RKN-susceptible tobacco variety W38 after inoculated with Pre-J2 *M. incognita* 14 days to 21 days. Female indicates female *M. incognita* isolated from tobacco roots after 30 days of inoculation with Pre-J2 *M. incognita*. (**B**–**F**) Life stage expression of the five effectors. The relative expression levels of *MiGST_N_4*, *MiCal*, *MiEFh*, *MiACPS*, and *MiTSPc* were quantified using quantitative RT-qPCR of four different *M. incognita* life stages: pre-J2, pra-J2, J3/J4, and female. The actin gene was used as a reference gene. The values were calculated using the 2^−^^△△*Ct*^ method and presented as the fold-change in mRNA level in various nematode developmental stages relative to that of pre-J2. Means ± SD were shown. The results shown are representative of at least three independent experiments. Different letters indicate significant differences based on one-way analysis of variance (ANOVA) followed by Duncan’s multiple range test (*p* < 0.05).

The newly hatched pre-J2 of *M. incognita* were inoculated with RKN-susceptible tobacco variety W38, and the nematodes were extracted at different developmental stages (Figure 5A). The expression of each effector gene at different developmental stages was analyzed by RT-qPCR. The results showed that the effector *MiGST_N_4* was barely expressed before inoculation and was strongly expressed immediately after inoculation. After that, its expression significantly increased at the parasitic third-stage juveniles (J3)/parasitic fourth-stage juveniles (J4) stage, but decreased at female stage. *MiCal* and *MiEFh* genes showed similar expression trends, with weak expression before inoculation, and were increased at the parasitic second-stage juveniles (Pra-J2) stage after inoculation, and continued at the J3/J4 stage, peaking at the J3/J4 stage, after which their expression began to decline at the female stage, but was still higher than at the pre-parasitic second-stage juveniles (pre-J2) stage before inoculation. The expression of the *MiACPS* effector gene was almost absent in the pre-J2 stage before inoculation, was up-regulated after inoculation and reached a peak point in the J3/J4 stage, then decreased significantly in the female stage, but the expression was almost the similar as that in the Pra-J2 stage. The effector *MiTSPc* was barely expressed at the pre-J2 stage before inoculation, its expression started to increase after inoculation and continued to the female development stage (Figure 5).

### 2.5. Effectors Can’t Induce Defense-Related Cell Death in the RKN-Susceptible Tobacco Variety W38

**Figure 6:**
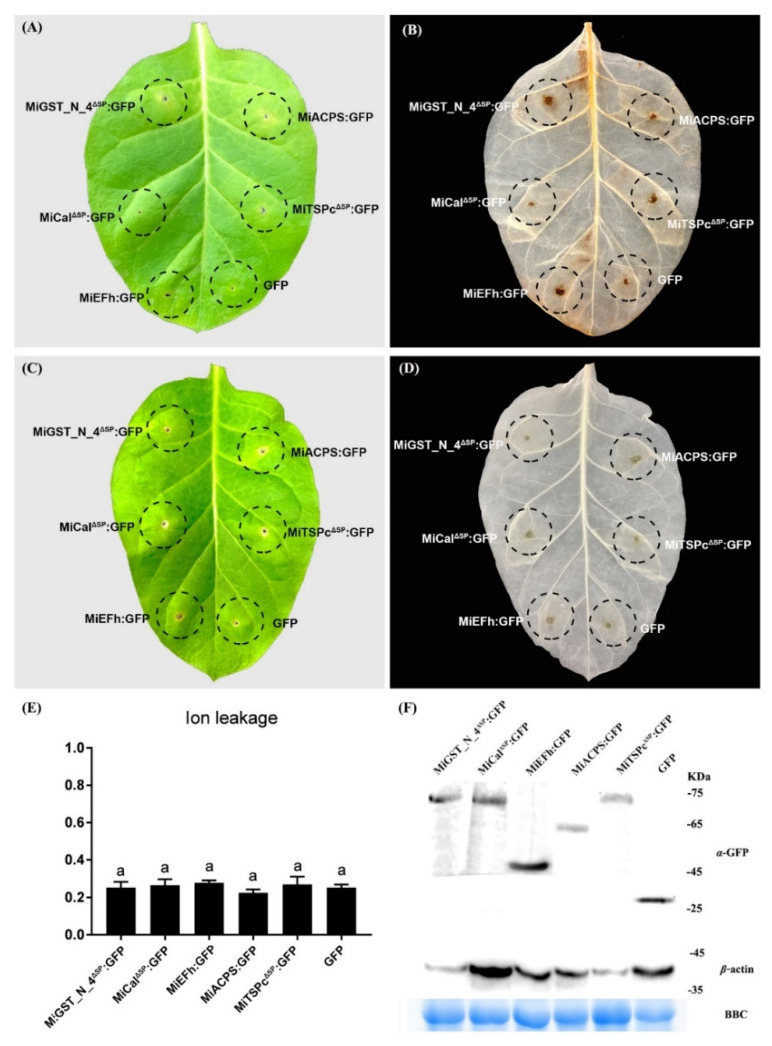
Analysis of reactive oxygen species (ROS) and hypersensitive response (HR) after inoculation of five effectors in the leaves of RKN-susceptible tobacco variety W38. The experiment was repeated at least three times, and each assay consisted of at least five plants with three leaves inoculated similarly. (**A**,**C**) The left panel shows a representative leaf of *A. tumefaciens* GV3101 carrying the vectors of MiGST_N_4^ΔSP^: GFP, MiCal^ΔSP^: GFP, MiEFh: GFP, MiACPS: GFP and MiTSPc^ΔSP^: GFP constructs injected into the leaves RKN-susceptible tobacco variety W38. Five representative leaves were photographed after 5 days. OD_600_ = 1. (**B**) shows leave cleared with 3,3′-diaminobenzidine blue (DAB). The brown area represents H_2_O_2_ localization. (**D**) Leaves cleared by ethanol injected with effectors after 5 days. (**E**) Ion leakage (mean ± SE, *n* > 5) was measured after injecting the effector 72 h. Error bars represent ± SD of three independent experiments. Letters on the bars indicate variability between samples (*p* < 0.05). Different letters indicate significant differences based on one-way analysis of variance (ANOVA) followed by Duncan’s multiple range test. (**F**) Normal expression and accumulation of all tested proteins in leaves as indicated by Western blotting analysis. Coomassie brilliant blue (CBB) staining was used to show equal up-sampling of protein samples.

Leaf transient expression was used to study the effector functions in plant-nematode interactions. In particular, it was used to investigate their roles in the elicitation of defense signaling [53]. Since the expression of different effectors at different development stages was not the same, we analyzed whether these effectors could induce defense responses or not. The results showed that after 5 days of transient expression of effectors in the RKN-susceptible tobacco variety W38, trace amounts of ROS remained in the leaf expression region for five effectors, and only faint traces of ROS could be observed, with the MiCal effector no traces of ROS were observed (Figure 6A,B). None of the five effectors induced HR in the RKN-susceptible tobacco variety W38 and only traces of pinholes could be observed (Figure 6C,D). Ion leakage analysis of the injected area showed that all effectors have similar effects as the control (Figure 6E). Western blotting data showed that all five effector fusion proteins were expressed in the RKN-susceptible tobacco variety W38 leaves (Figure 6F).

### 2.6. Effectors Trigger Defense-Related Cell Death in the RKN-Resistance Tobacco Variety K326

**Figure 7:**
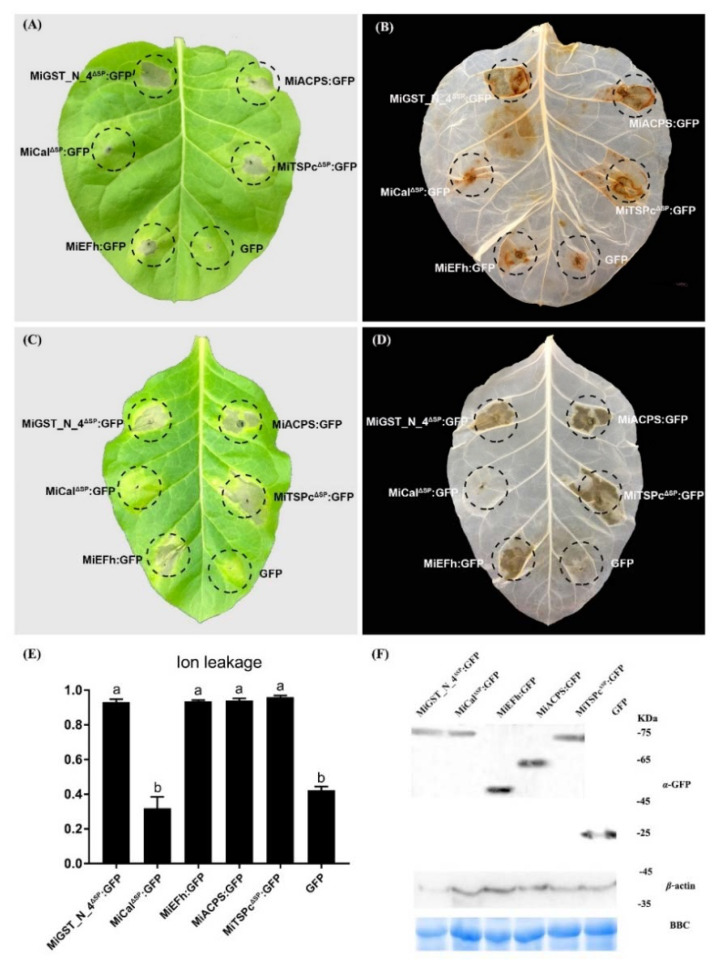
Analysis of ROS and HR after inoculation of five effectors in the RKN-resistance tobacco variety K326. The experiment was repeated at least three times, and each assay consisted of at least five plants with three leaves inoculated similarly. (**A**,**C**) shows a representative leaf of *A. tumefaciens* GV3101 harboring the vectors of MiGST_N_4^ΔSP^: GFP, MiCal^ΔSP^: GFP, MiEFh: GFP, MiACPS: GFP, and MiTSPc^ΔSP^: GFP constructs injected into the RKN-resistance tobacco variety K326. The HR occurred in the leaf at the injection position of 5 days after inoculation with the effector. Five representative leaves were photographed after 5 days. OD_600_ = 1. (**B**) Leaves cleared with 3,3′-diaminobenzidine blue (DAB). The brown area represents H_2_O_2_ localization. (**D**) leaves cleared by ethanol injected with effectors after 5 days. (**E**) Ion leakage (mean ± SE, *n* > 5) was measured 72 h after effector injection in the RKN-resistance tobacco variety K326. Error bars represent ± SD of three independent experiments. Letters a-b on the bars indicate significant differences (*p* < 0.05) between samples. Different letters indicate significant differences based on one-way analysis of variance (ANOVA) followed by Duncan’s multiple range test. (**F**) Normal expression and accumulation of all tested proteins in plant cells as indicated by Western blotting analysis. Coomassie brilliant blue (CBB) staining was used to show equal up-sampling of protein samples.

Since the five effectors caused weak ROS accumulation in the RKN-susceptible tobacco variety W38 but could not induce HR, we tested whether those effectors could trigger ROS and HR in the RKN-resistance tobacco variety K326. The results showed that MiGST_N_4, MiEFh, MiACPS, and MiTSPc caused cell necrosis, and strong ROS could be observed at the injection sites (Figure 7A,B). The results of HR were similar to those of ROS; for instance, MiGST_N_4, MiEFh, MiACPS, and MiTSPc caused significant HR, while MiCal did not cause any HR (Figure 7C,D). Ion leakage assay of the injected region showed that the ion permeation rates of MiGST_N_4, MiEFh, MiACPS, and MiTSPc were significantly higher than the control, while the ion permeation rates of MiCal effectors were not different from the control (Figure 7E). Western blotting data showed that all effector fusion proteins accumulated in the RKN-resistance tobacco variety K326 leaves (Figure 7F). These results indicate that MiGST_N_4, MiEFh, MiACPS and MiTSPc can elicit the ROS burst, strong HR and significant ion leakage levels in resistant tobacco plants.

## 3. Discussion

Root-knot nematodes spend most of their life cycle inside the host root and feed on living tissue. To promote parasitism, a variety of effectors are secreted to degrade plant cell walls, absorb nutrients, manipulate defense systems, and establish feeding sites [54,55]. The genome of *M. incognita* analysis revealed that there are about 10,000 proteins; the function of most of these proteins is unknown [56]. Current studies mainly focused on the species with signal peptides at the N-terminal. However, only about 10% of the proteins in *M. incognita* contain signal peptides, and most effectors do not contain any signal peptides [57]. Few studies have been conducted on these effectors that do not contain signal peptides. In this study, we obtained five *M. incognita* effector proteins that may interact with PsoRPM3 protein by IP and LC-MS in *P. sogdiana.* The five effectors were named MiCal, MiGST_N_4, MiTSPc, MiEFh, and MiACPS, and their molecular weights and structures differed significantly. MiCal, MiGST_N_4, and MiTSPc effectors have signal peptides at the N-terminus, while MiEFh and MiACPS effectors do not have signal peptides, indicating that the effectors associated with PsoRPM3 are also diverse.

Root-knot nematodes secrete effectors mainly through the three specialized secretory oesophageal glands, one DG, and two SvG [54,58]. Generally, the SvG play a major role during the nematode invasion and migration stages. The cells of the SvG are more active before nematode invasion but begin to atrophy and become smaller during the later stages of parasitism after nematode fixation. In contrast, the DG begins to grow longer after parasitization and plays a major role in the formation and maintenance of feeding sites [59]. During the migration process, nematodes secrete effectors from apoplasts to plant tissues via stylets [60,61]. Therefore, precise positioning of effectors can provide a better understanding of their biological functions. In this study, we showed that *MiCal*, *MiGST_N_4*, *MiEFh*, and *MiACPS* effectors were secreted in the SvG of pre-J2 *M. incognita*, whereas MiTSPc effectors were secreted in the DG, suggesting that they play different roles during parasitism. Expression analysis of five effectors at different developmental stages of nematodes showed that *MiGST_N_4*, *MiCal*, *MiEFh* and *MiACPS* effectors secreted in the SvG had higher expression levels at the pra-J2 stage and J3/J4 stage, but significantly lower expression at the female stage, indicating that these four effectors secreted in the SvG are necessary in the early stage of parasitism. The effector *MiTSPc* is secreted by the DG, and its expression was highest in the female stage, suggesting that effectors MiTSPc may play a role in the formation and maintenance of feeding sites in the late parasitic stage.

Root-knot nematode effectors entering host tissues can promote parasitism and change plant functions by binding to their proteins [60,62]. For instance, the MiMIF-2 effector of *M. incognita* disrupts plant cell metabolism and the SA-mediated immune response through enzymatic activity [63]. MiISE5 regulates multiple signaling pathways in the early stage of parasitism and suppresses the host’s resistance response [25]. In addition, the Mi-CRT, MjTTL5, and MiMsp40 effectors of *M. incognita* have shown similar functions [27,39,64]. Inhibition of plant immune responses by these effectors generally requires targeting the host cell nucleus and disrupting plant immunity via alteration of the plant cell cycle through the manipulation of processes [65,66]. The 7H08 effector of *M. incognita* has transcriptional activity in plants, but the target gene in the host has not been determined yet [67]. The 16D10 effector gene of *M. incognita* targets Scarecrow-like transcription factors [68]. Thus, effectors with nuclear localization signals are thought to play a critical role in the regulation of plant immune responses. This study found five effectors, located in the membrane and nucleus of plant cells having different intensity of fluorescence signals. The fluorescence signals of MiCal, MiEFh and MiACPS were stronger in the cell membrane and nucleus; MiGST_N_4 had a stronger signal in the nucleus than in the cell membrane, and MiTSPc had a weaker signal in both the nucleus and the cell membrane, suggesting that these five effectors may have different roles in the parasitic process. There was almost no ROS production in the RKN-susceptible tobacco variety W38, and no HR at all. In contrast, four effectors (MiGST_N_4, MiEFh, MiACPS, and MiTSPc) in the RKN-resistance tobacco variety K326 elicited HR and produced a strong ROS accumulation, indicating that these four effectors may be involved in triggering the immune response of the host plant.

## 4. Materials and Methods

### 4.1. Plant Materials and Nematodes

Seeds were sown using the RKN-susceptible tobacco variety W38 and the RKN-resistance tobacco variety K326 carrying the gene *Rk1* resistant to *M. incognita* [69]. The plants grown at 28 °C under a 16 h light/8 h dark cycle. One-month seedlings were inoculated with *M. incognita* infective juveniles. *N. benthamiana* was used for subcellular localization experiments 3 weeks after sowing.

We followed the method reported by Huang et al. [70] to collect the *M. incognita* at different developmental stages. Briefly, we rinsed the RKN-susceptible tobacco variety W38 tobacco roots that had been inoculated with *M. incognita* after 7, 14, 21, and 30 days with tap water, cut them into ~1 cm sticks, immersed roots in the Pectinex and Celluclast mix, incubated the mixture for 4 h (overnight) at RT under gentle agitation (orbital shaker, 50 rounds per minute), and purified the nematodes by sucrose gradient centrifugation. According to the report on the life history of *M. incognita* by Abad et al. [57], the Par-J2, J3/J4 and females were collected with a pipette under a stereo microscope. In addition, newly hatched pre-J2 of *M. incognita* were used for in situ hybridization and RNA extraction for gene cloning.

### 4.2. PsoRPM3 Protein Immunoprecipitation (IP) Experiments and LC-MS Analysis

PsoRPM3 protein IP experiments and LC-MS analysis was conducted according to the method reported by Xiao et al. [71]. Briefly, the PsoRPM3 carrying the MBP tag protein was constructed into the prokaryotic expression vector pMal-c2x, and the fusion protein MBP-PsoRPM3 was induced to express overnight at 37 °C and 180 rpm. After sonication, the supernatant was collected and the fusion protein was collected using MBP beads (New England Biolabs, Ipswich, MA, USA). The total protein derived from the root inoculated with 10,000 pre-J2 of *M. incognita* after 3 days, and the total protein of roots that had not been inoculated with *M. incognita* were used as controls. Incubate in a shaker at 4 °C for 2 h. After centrifugation and elution, the protein was sent to Beijing Qinglian Biotech Co., Ltd. for the LC/MS tests. The acquired mass spectrometric data were analyzed using ProteomeDiscoverer2.1 and then anatomized for searches of the NCBI (https://www.ncbi.nlm.nih.gov, accessed on 12 September 2019) database and *M. incognita* (https://www6.inrae.fr/meloidogyne_incognita/, accessed on 15 September 2019) database.

### 4.3. Gene Amplification and Sequence Analysis

Total RNA was extracted from *M. incognita* different developmental stages collected, as described above, by Trizol (Invitrogen, Waltham, MA, USA). First-strand cDNA was synthesized using the Novo Script Plus All-in-one 1st strand cDNA synthesis SuperMix (Novoprotein Scientific Inc, Shanghai, China). To clone the effector gene sequence, primers covering the whole sequence were designed to perform PCR from cDNA templates. For PCR amplification, 200 ng of cDNA template was used in a 50 µL reaction mixture consisting of 2 × PCR buffer for KOD FX, 2 mM dNTPs, 10 pM for each primer, and 1.0 U KOD FX (TOYOBO Co., Ltd., Kita-ku, Osaka, Japan). All primers were synthesized by Sangon Biotech Co., Ltd. (Shanghai, China) and are listed in Supplementary Table S1.

To analysis protein sequences of the effectors, SignalP 5.0 (www.cbs.dtu.dk/services/SignalP/, accessed on 20 November 2019), SMART (http://smart.embl-heidelberg.de/, accessed on 22 November 2019), ExPASy (https://web.expasy.org/protparam/, accessed on 20 November 2019) and Motif Scan (https://myhits.sib.swiss/cgi-bin/motif_scan, accessed on 25 November 2019) were used to predict potential traits of effector proteins.

### 4.4. In Situ Hybridization of Effectors in Pre-J2

In situ hybridization was performed using digoxigenin-labeled cDNA probes on freshly hatched pre-J2 of *M. incognita* to detect the expression and secretion sites of effectors [72]. Targeted effector fragments were amplified using specific primers (Supplementary Table S1). PCR products were used as templates for asymmetric PCR using the MyLab^TM^ Digoxin Labeling and Hybridization Detection Kit (Beijing Merab Medical Technology Co., Ltd., Beijing, China) to synthesize DIG-labeled sense and antisense cDNA probes. A total of 10,000 newly hatched pre-J2 of *M. incognita* were collected, fixed, transparent, and hybridized according to the method reported by Jaouannet et al. [73]. Hybridization probes were immunodetected with anti-DIG-AP enzyme complexes followed by NBT/BCIP chromogenic substrates. The hybridized nematodes were observed and photographed with an Axio Imager M2 microscope.

### 4.5. Developmental Expression Analysis

RNA samples were extracted from pre-J2, pra-J2, J3/J4 and Female stages isolated from the RKN-susceptible tobacco variety W38 as described above. The cDNA was synthesized using TransScript All-in-One First-Strand cDNA Synthesis SuperMix (Transgen Biotech, Beijing, China). RT-qPCR was conducted in an ABI Prism 7000 real-time PCR system (Applied Biosystems, Forster City, CA, USA). Root-knot nematode actin protein was used as an internal reference. PCR was performed using the SuperReal PreMix Plus (Tiangen Biotech Co., Ltd., Beijing, China) kit with a PCR program of 30 s at 95 °C for 40 cycles, followed by 5 s at 95 °C, 31 s at 60 °C, and 15 s at 95 °C. The results were analyzed using the 2^−ΔΔ*Ct*^ method [74] for data analysis. Three technical replicates and independent experiments replicates were performed for each reaction in all experiments.

### 4.6. Subcellular Localization

*A. tumefaciens* strains of GV3101 harboring the construct of 35S: MiCal^ΔSP^: GFP, 35S: MiGST_N_4^ΔSP^: GFP, 35S: MiTSPc^ΔSP^: GFP, 35S: MiEFh: GFP, 35S: MiACPS: GFP were suspended in infiltration buffer to an OD_600_ of 1 and were syringe-infiltrated to into the leaves of three- to four-week-old *N. benthamiana* plants as described by Zhao et al. [31]. The strain GV3101 carrying 35S: GFP was used as a control. Leaf tissue collected 48 h after infiltration was visualized under a confocal laser-scanning microscope (Zeiss LSM 880) at an excitation wavelength of 488 nm and emission was detected at 495–530 nm [75].

### 4.7. Effector Virulence Testing

To test whether the effectors could trigger cell death, an assay for triggered cell death was performed in tobacco (the RKN-susceptible tobacco variety W38 and the RKN-resistance tobacco variety K326). The cultured *A. tumefaciens* strains of GV3101 carrying the construct of effectors as above (OD_600_ = 1) were initially infiltrated into the tobacco leaves. Simultaneously, the GFP gene was expressed alone as a control. The leaves were monitored for symptoms, and photographs were acquired 5 days after infiltration. The experiment was repeated at least three times, and each assay consisted of at least five plants with three leaves inoculated similarly.

### 4.8. H_2_O_2_ Detection

H_2_O_2_ was detected according to previous studies [76,77]. Briefly, incubated tobacco leaves were placed in 3,3′-diaminobenzidine (DAB)-HCI (Beijing Nobled Technology Co., Ltd., Beijing, China) (1 mg/mL^−1^, pH 3.8) solution and incubated for 8 h. Leaves were cleared in boiling 96% ethanol for 10 min and stored in 96% ethanol for visualizing.

### 4.9. Electrolyte Leakage Detection

Ion leakage was measured as described previously [78]. Briefly, five-leaf discs (1.2 cm diameter) were harvested from tobacco leaves post-agroinfiltration and put in 5 mL of sterile H_2_O_2_ in a 15-mL polypropylene tube. The samples were shaken at room temperature for 3 h, and conductivity (C1) was measured by a conductivity meter (Model 4403, Markson Science, Inc., Del Mar, CA, USA). Samples were then boiled for 15 min, and the conductivity was measured again as the C2 value. Ion leakage was calculated as C1/C2 ratio. All assays were repeated three times. Statistical analyses were performed using GraphPad Prism 7.0 (GraphPad Software, San Diego, CA, USA).

### 4.10. Protein Extraction and Western Blot Analysis

*A. tumefaciens* GV3101 strain expressing GFP-epitope tagged proteins was infiltrated into tobacco (the RKN-susceptible tobacco variety W38 and the RKN-resistance tobacco variety K326) for expression. Leaf tissue was collected 36 h after infiltration and stored at −80 °C until further use. Tobacco leaf tissue was ground with liquid nitrogen and with lysis buffer (10 μL 100 mM PMSF, 1 μL Cocktail, 14 μL 10% Tralatone, and 1 mL protein extraction buffer; Huaxing Boltons, Beijing, China), and centrifuged at 12,000× *g* for 25 min. Proteins were separated by SDS-PAGE in an 8% gel. Western blotting was carried out using the semidry method on polyvinylidene difluoride (PVDF) membranes. Blots were probed with mouse anti-GFP antibodies (Pao Yijie Technology Co., Beijing, China) at a dilution of 1:5000. The secondary goat-anti-mouse antibody (HRP) was used at a dilution of 1: 5000. The signal was visualized with the eECL Western Blot Kits (Kangwei Century Biotechnology Co., Ltd., Beijing, China) according to the manufacturer’s instruction.

## Supplementary Materials

The following supporting information can be downloaded at: https://www.mdpi.com/article/10.3390/ijms23031498/s1.
