# Supplementary material for: Characterization of Five Meloidogyne incognita Effectors Associated with PsoRPM3
Source: Int J Mol Sci. 2022 Jan 28;23(3):1498. doi: 10.3390/ijms23031498 (PMC8836280; doi:10.3390/ijms23031498)
Supplement: Supplementary file 1 [file ijms-23-01498-s001.zip › ijms-1514290-supplementary.pdf]

Characterization of Five *Meloidogyne incognita* Effectors Associated with PsoRPM3

Wenjiang Pu †, Kun Xiao †, Sifang Luo, Haifeng Zhu, Zizhen Yuan, Chaoyuan Gao and Jianfang Hu *

Table S1. PsoRPM3's IP result.

Protein Accession Number (in NCBI Database)	Protein Accession Number (in *Meloidogyne incognita* Database)	Types of Nematodes	Signal Peptide	Molecular Weight (KD)	Fasta Headers	LFQ Intensity CK	LFQ Intensity CL
AHB20148.1;AAO16990.1;AAO16989.1	Non	*Meloidogyne enterolobii*			>AHB20148.1 actin	0	39731000
AHX24635.1;AHX24634.1;AHX24633.1;AHX24632.1	Minc3s00127g05433	*Meloidogyne incognita, Meloidogyne hispanica, Meloidogyne arenaria, Meloidogyne javanica, Meloidogyne hapla*	Yes	46.35	>AHX24635.1 calreticulin, partial; >AHX24634.1 calreticulin, partial; >AHX24633.1 calreticulin, partial; >AHX24632.1 calreticulin, partial	0	5696900
AAN15809.1	scaffold166774_cov71.g22242	*Meloidogyne incognita*	Yes	45.86	>AAN15809.1 putative esophageal gland cell secretory protein 29	0	3862500
ACI43068.1	Non	*Capsicum annuum*			>ACI43068.1 NBS-LRR root-knot nematode resistance protein	0	3651700
AAV87529.1	Minc3s00081g03887	*Meloidogyne incognita*	Yes	40.99	>AAV87529.1 chymotrypsin-like serine proteinase	0	2533800
AHG55039.1	Non	*Meloidogyne enterolobii*			>AHG55039.1 heat shock protein 70	0	1834000
YP_009029729.1;AHY20093.1	Non	*Meloidogyne chitwoodi*			>YP_009029729.1 NADH dehydrogenase subunit 3 (mitochondrion); >AHY20093.1 NADH dehydrogenase subunit 3 (mitochondrion)	0	1035400
YP_009040641.1;CDI40117.1;AUX81265.1;AHN16445.1	Non	*Meloidogyne graminicola*			>YP_009040641.1 NADH dehydrogenase subunit 6 (mitochondrion); >CDI40117.1 NADH dehydrogenase respiratory chain subunit 6 (mitochondrion); >AUX81265.1 NADH dehydrogenase subunit 6 (mitochondrion)	0	798410
sp|Q9ZSK2.1|ADF6_ARATH	Non	*RecName*			>sp|Q9ZSK2.1|ADF6_ARATH RecName: Full=Actin-depolymerizing factor 6; Short=ADF-6; Short=AtADF6	0	653130
AHN52302.1	Minc3s00642g15511	*Meloidogyne incognita*	No	19.96	>AHN52302.1 programmed cell death protein 6	0	478740

ACF71478.1;ACF71476.1;ACF71475.1;ACF71474.1;ACF71468.1;ACF71477.1;ACF71473.1;ACF71472.1;ACF71471.1; ACF71470.1;ACF71469.1;ACF71467.1;ACF71466.1;ACF71465.1;ACF71464.1;ACF71463.1;ACF71462.1;CBA18120.1; CBA18119.1;CBA18118.1;CBA18117.1	Non	*Meloidogyne hapla, Meloidogyne javanica, Meloidogyne arenaria*			>ACF71478.1 major sperm protein, partial; >ACF71476.1 major sperm protein, partial; >ACF71475.1 major sperm protein, partial; >ACF71474.1 major sperm protein, partial	0	425760
AFG25450.1	Minc3s01334g22880	*Meloidogyne incognita*	No	35.86	>AFG25450.1 t04g9.4, partial [Meloidogyne incognita]	0	318720
ALG00081.1	Non	*Purpureocillium lilacinum*			>ALG00081.1 glycoside hydrolase family 31	0	242300
ATO58929.1;ATO58928.1;ATO58916.1;ATO58936.1;ATO58935.1;ATO58930.1;ATO58921.1; ARU79877.1	Non	*Meloidogyne enterolobii*			>ATO58929.1 cytochrome oxidase subunit 1, partial (mitochondrion); >ATO58928.1 cytochrome oxidase subunit 1, partial (mitochondrion); >ATO58916.1 cytochrome oxidase subunit 1, partial (mitochondrion)	0	132620
CAU15484.1;CAU15486.1	Non	*Meloidogyne artiellia*			>CAU15484.1 heat shock protein 90 [Meloidogyne artiellia]; >CAU15486.1 heat shock protein 90 [Meloidogyne artiellia]	0	104070
AAY84711.2	Minc3s07235g40885	*Meloidogyne incognita*	No	179.48	>AAY84711.2 dual oxidase	0	46631
ADM16640.1	Non	*Meloidogyne javanica*			>ADM16640.1 putative transmembrane cation transporter	0	35593

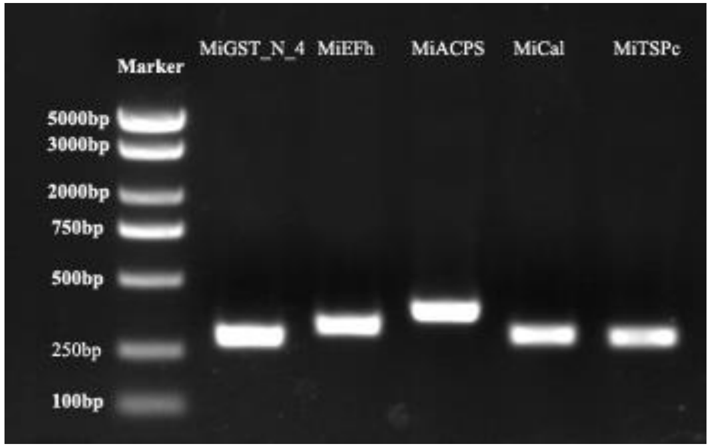

Figure S1. In *situ* probe primer specific detection of all effectors. Through gel electrophoresis, a single target amplified by the in *situ* probe primers of MiGST_N_4 (288bp), MiEFh (354bp), MiACPS (388bp), MiCal (304bp), MiTSPc (302bp) was detected between 250 bp and 500 bp.
